# Let Us Not Forget About Bleeding: A Case Report and Brief Literature Review on Hemorrhagic Vestibular Schwannoma

**DOI:** 10.7759/cureus.32269

**Published:** 2022-12-06

**Authors:** Francisco Alves de Sousa, Ângela Reis Rego, Armindo Picão Fernandes, Ana Pinto, Luís Meireles

**Affiliations:** 1 Otolaryngology - Head and Neck Surgery, Centro Hospitalar Universitário do Porto, Porto, PRT; 2 Neurosurgery, Centro Hospitalar Universitário do Porto, Porto, PRT; 3 Otolaryngology, Centro Hospitalar Universitário do Porto, Porto, PRT

**Keywords:** vestibular schwannoma, neuro-otology, otolaryngology case report, neuro-surgery, rare presentation, benign brain tumor, post-hemorrhagic hydrocephalus, hidrocephalus, risk of bleeding, intracranial hemorrhage (ich)

## Abstract

Hemorrhagic vestibular schwannoma (HVS) consisting of acute intratumoral and subarachnoid hemorrhage is a rare phenomenon. We present the case of a 31-year-old woman who attended the Otorhinolaryngology department with right-sided intense tinnitus, dizziness, imbalance, and headache. Brain computed tomography revealed a spontaneous hyperdensity in the posterior fossa with marked deformation of the brainstem, middle cerebral peduncle, and cerebellum, with the near collapse of the fourth ventricle. Ophthalmology evaluation confirmed bilateral papilledema. Brain magnetic resonance imaging confirmed a voluminous 33 x 28 x 29 mm extra-axial lesion centered on the right pontine-cerebellar angle cistern, extending from the plane of the trigeminal nerve/tent of the cerebellum. The acoustic pore was enlarged. The patient underwent retrosigmoid craniotomy and microscopic tumor resection showing significant improvement in the follow-up. Pathological findings confirmed HVS. Delayed treatment of HVS can increase morbidity or even be fatal. The objective of this work is to describe and revise HVS, in order to bring awareness to this uncommon entity.

## Introduction

Vestibular schwannoma (VS) is a benign, slow-growing tumor that derives from Schwann cells [1,2]. It originates from the vestibulocochlear nerve at the level of the internal auditory canal (IAC) and can reach the pontocerebellar angle (PCA). It represents about 90% of all PCA tumors [3,4]. Unilateral hearing loss with or without tinnitus is the most commonly reported symptom, while sudden deafness and acute vertigo are rarer possible presentations [3-5]. Hemorrhagic VS (HVS) consists of acute intratumoral and/or subarachnoid hemorrhage (SAH) and is a rare phenomenon [6]. HVS may present with a myriad of symptoms that are not so frequently observed in non-hemorrhagic VS. It is important to note that these cases may not attend to neurology or neurosurgical emergency rooms, but instead to Otorhinolaryngology ones, such as the case here presented, due to the type of commonly involved complaints in HVS. The case here presented illustrates the importance of a timely diagnosis in HVS and the articulation with the Neurosurgery team, in order to prevent complications while providing the best standard of care. The ultimate purpose of this case description is to raise awareness among attending physicians to the possibility of serious complications deriving from a well-known “benign” lesion such as VS. This case is unique in the way it presented to the Otorhinolaryngology clinic, without limitation or obvious neurological signs that could easily raise suspicion of an intracranial hemorrhage.

## Case presentation

We illustrate the case of a HVS, starting from the first evaluation in the emergency department to surgical approach. A 31-year-old woman attended the Otorhinolaryngology emergency department complaining about acute debilitating right-sided intense tinnitus, dizziness, imbalance, and headache that began the day before making her search for medical attention. She also reported one episode of projectile vomiting earlier in the morning. When questioned, also referred to periodic blurred vision in the last 2 months, which had worsened in the past week with higher frequency and duration of paroxysms. When explored, she described horizontal diplopia with a conjugated gaze to the right. 

**Figure 1:**
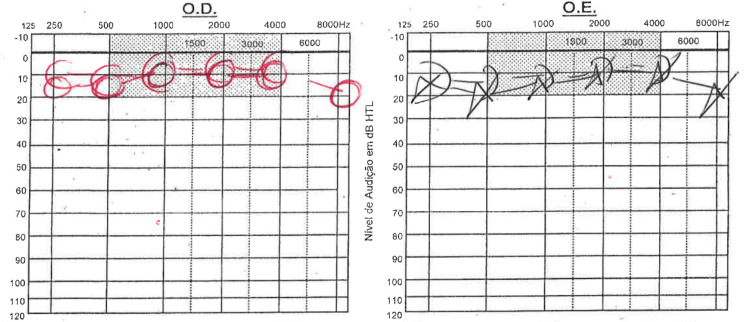
Audiogram performed two years before patient´s admission. This formerly available audiogram was unremarkable.

She had been referred to an Otorhinolaryngological evaluation two years before due to right-sided aural fulness complaints with occasional dizziness. At that time, she had an audiogram done that was unremarkable (Figure 1). She had no other known past history of disease and reported herself as healthy otherwise.

**Figure 2:**
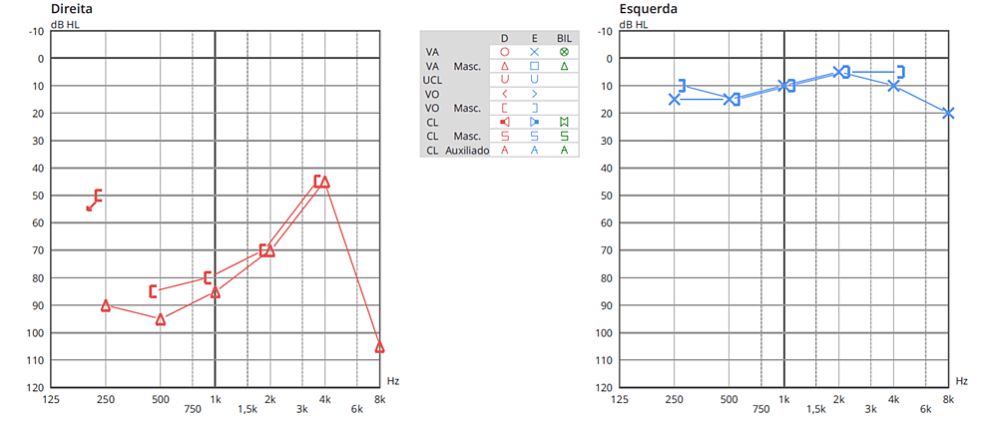
Audiogram at admission. Severe right-sided neurosensorial hearing loss on lower frequencies was found on admission.

At observation, the patient was fully awake and vigilant showing a Glasgow coma scale of 15. She was oriented in space, time and person. Otoscopy was normal bilaterally. There was a negative Rinne’s test in the right ear with weber´s lateralization to the left, a gaze-evoked nystagmus with right-sided fast phase, a discrete limitation on the right eye abduction, positive head impulse test to the right and a right-sided peripheral grade III House-Brackmann facial palsy. No clear additional motor or sensory deficits were present. There was no dysmetria in the finger-to-nose or heel-to-shin test. It was not possible to test Romberg or Unterberger due to considerable imbalance. An audiogram was performed which revealed a severe neurosensorial hearing loss on lower frequencies (Figure 2) and a tone decay of 50 dB (limit of the audiometer) after 10 s at 2 kHz.

**Figure 3:**
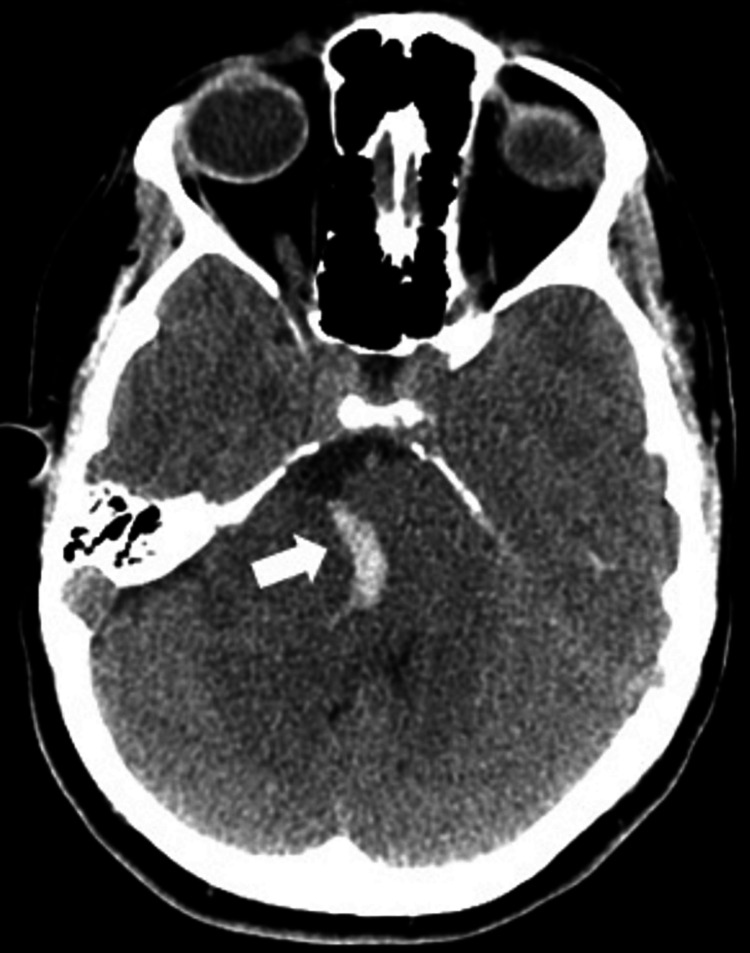
Axial pre-operative CT scan. Axial brain CT scan slice revealing spontaneous hyperdensity in the posterior fossa, suggestive of acute hemorrhage. Marked deformation of the brainstem, middle cerebral peduncle and cerebellum, with shift to the left and near collapse of the fourth ventricle.

These findings raised suspicion for a central inflammatory or tumoral etiology; hence, urgent brain imaging was requested. A brain computed tomography scan revealed a spontaneous hyperdensity in the posterior fossa, suggestive of an acute hemorrhagic event of a PCA tumor (Figure 3). No apparent SAH was present. The brainstem and the fourth ventricle were compressed by mass effect. There was no clear dilation of the third and lateral ventricles/concurrent ventriculomegaly, but further ophthalmology evaluation unveiled bilateral papilledema.

**Figure 4:**
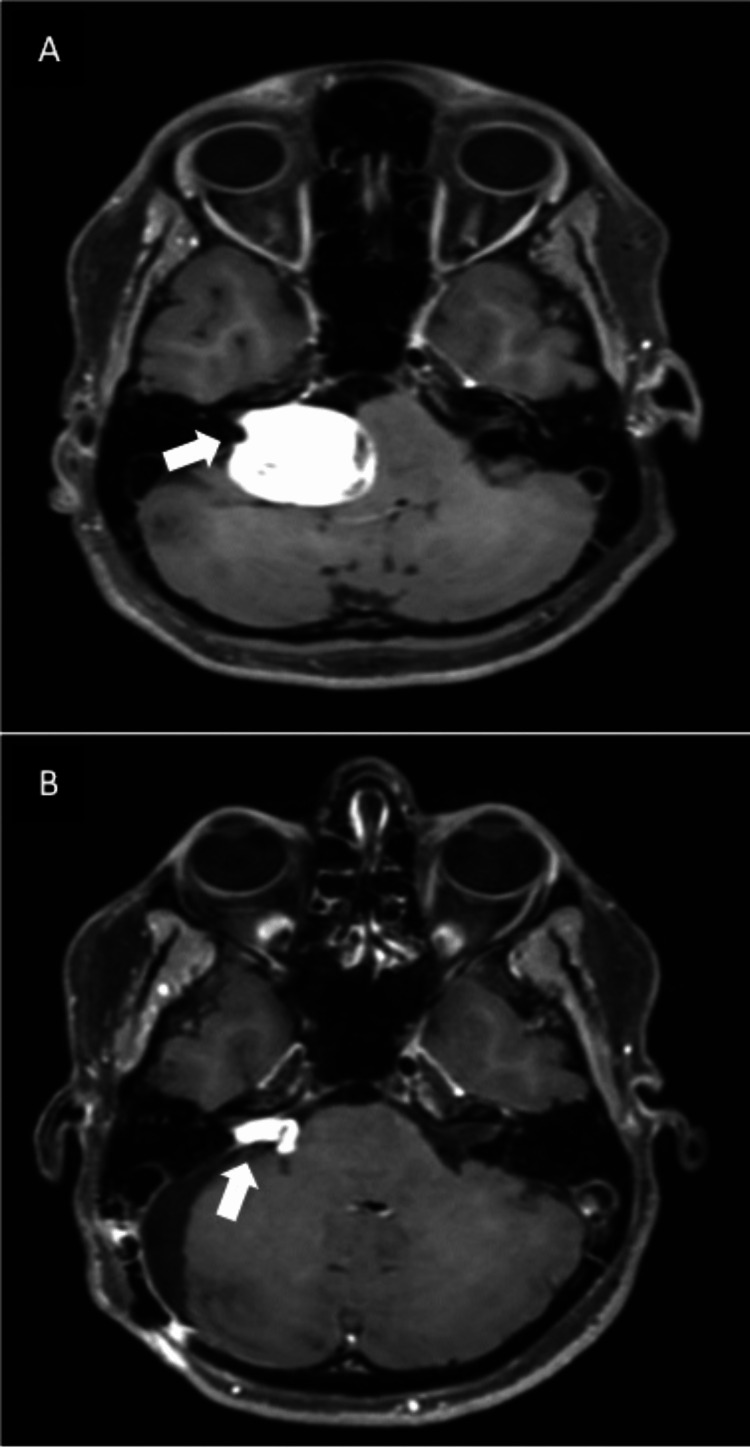
Pre-operative and post-operative MRI. (A) Preoperative axial MRI T1 gad imaging showing a voluminous extra-axial lesion centered on the right pontine-cerebellar angle cistern, extending from the plane of the trigeminal nerve/tent of the cerebellum to the plane of the jugular foramen, 33 x 28 x 29 mm anteroposterior, transverse and craniocaudal, respectively. The acoustic pore is enlarged. (B) Four months post-operative axial MRI in T1 Gad showed a right-sided tumoral residuum with intracanalicular and cistern components, 10 x 18 x 14 mm anteroposterior, transverse, and craniocaudal, respectively.

At this point, the patient was referred to the Neurosurgery team and brain magnetic resonance imaging was performed (Figures 4A, 4B) which confirmed the presence of a voluminous extra-axial lesion centered in the PCA with intracapsular bleeding. No associated aneurysm was noted in the contrasted brain magnetic resonance imaging.

**Figure 5:**
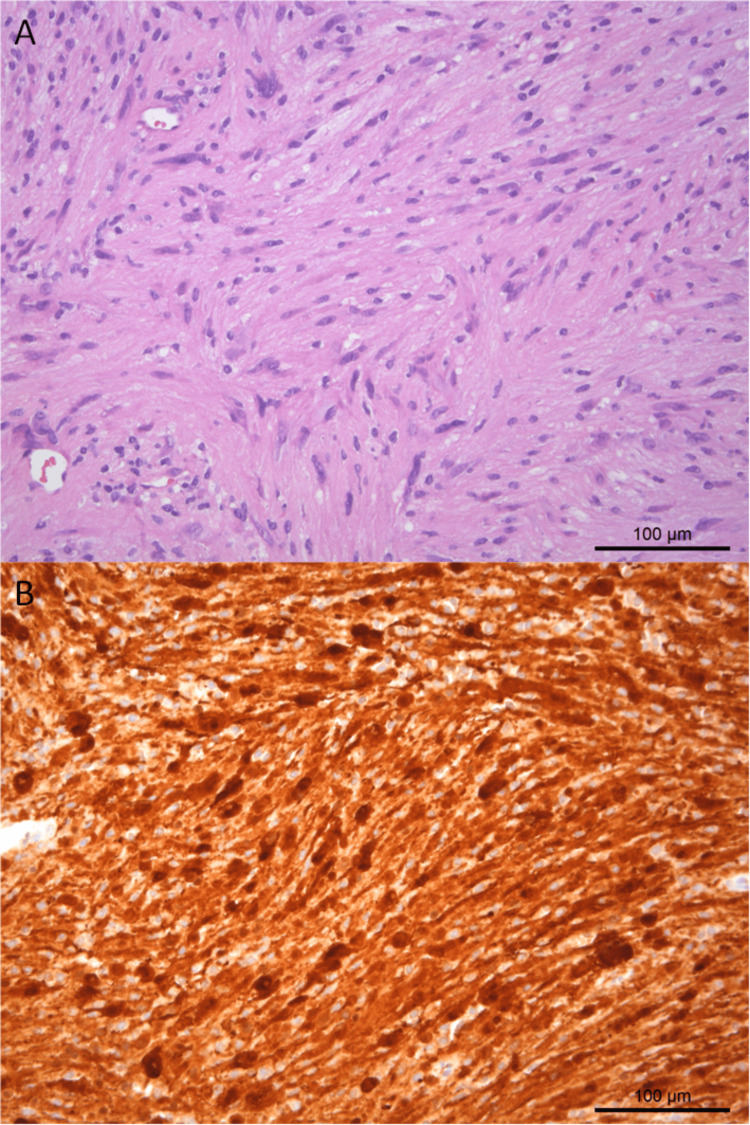
Anatomopathological analysis of the tumoral specimen. Neoplastic lesions composed predominantly of spindle-shaped nuclei cells arranged in fascicles (H&E) (A). The tumor cells are immunoreactive for S100 protein (B).

The patient´s symptoms were stabilized after a period of intravenous ondansetron and dexamethasone. She was brought to the operating room four days later after careful explanation about the benefits and risks of intervention and signing informed consent. A retrosigmoid craniotomy approach was used and intraoperative neuromonitoring was adopted in an attempt to preserve facial nerve functioning. Initial cerebrospinal fluid drainage from the Magna/peri-bulbar cisterns was effective, with consequent cerebellar relaxation. Subsequent tumor identification showed a whitish-yellow color and a well-defined capsule. After definition of a safe entry zone, capsule opening and tumor “debulking” with ultrasonic aspiration was performed - the tumor was soft (but not aspirable by regular suctioning). The dissection continued alternating peripheral arachnoid dissection with tumor debulking, while preserving both the arachnoid and capsular planes as much as possible, for greater safety. Drainage of the hematoma was performed in the deepest portion, close to the brainstem, and was intracapsular rather than SAH. Additional drilling of the acoustic pore allowed the exposure of the internal acoustic canal, which was invaded by the tumor. Removal of the intracanalar tumor portion was undertaken partially, as the tumor was adherent to the nerves and the facial nerve could not tolerate its manipulation in the monitoring. As pre-planned, the priority was to preserve the function of the facial nerve, so tumoral removal was done up to the limit of what was considered safe, based on anatomical references and facial nerve monitoring. This functional concerns implied leaving tumor residue along the path of the facial nerve. Residue/capsule was left in the upper and lower pole portion of the tumor and also at the interface with the brainstem - where the plane was worse, possibly related to the previous hemorrhage. Hence, a tumoral residuum was left in order to preserve anatomical integrity of cranial nerves, major local vessels and brainstem (Figure 4). Pathologic analysis of tumoral specimen sealed the diagnosis of schwannoma WHO grade I (Figures 5A, 5B).

**Figure 6:**
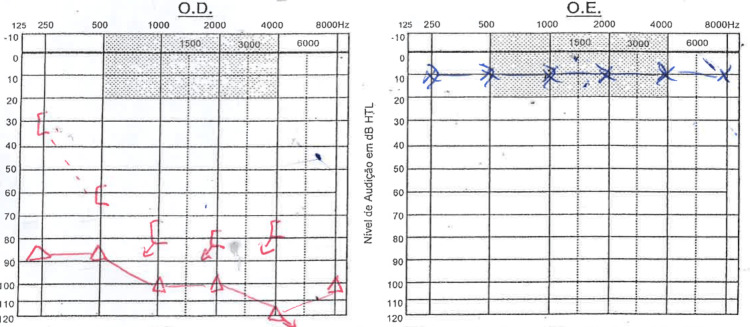
Audiometric follow-up six months after surgery.

The patient´s postoperative period was uneventful, with resolution of headache and progressive improvement. No new neurological deficits ensued besides the already installed facial palsy. Gait imbalance demanded a period of physical rehabilitation. The patient was discharged 15 days after surgery with regained autonomy in daily activities. At the six-month follow-up, the patient reported progressive improvement of facial palsy, with objective examination showing grade I House-Brackmann palsy. Considering hearing, the patient reported deafness in the right ear. Audiometrical analysis at this time of follow-up confirmed a functional cophosis in the right ear with total loss of speech discrimination (Figure 6).

## Discussion

In order to perform a literature review, a PubMed search was performed using the MeSH terms “vestibular schwannoma” and “hemorrhage.” Articles published between 1990 and 2022 were considered. Of all the intracranial hemorrhages, only between 1% and 11% correspond to hemorrhagic brain tumors, most commonly seen in higher WHO grade ones like glioblastoma multiforme [6,7]. Concerning SAH, only about 0.4 % arise from tumor hemorrhage [6]. VS incidence greatly varies with age, with a global estimated incidence of around 1.2 cases per 100,000 person-years in developed countries [8,9]. Based on research data, VS bleeding has an extremely low incidence of about 2.15 cases per year worldwide [6]. Thus, one can deduce that the vast majority of VS do not associate with bleeding at all. However, ITH in VS may be more prevalent than originally thought. A histological examination of 274 VS specimens indicated that almost all had different degrees of intratumoral microhemorrhage [10]. Indeed, tumoral hemorrhage is more common in the second most prevalent APC tumor (i.e., meningioma). In meningioma, as in VS, the audiovestibular symptoms are also common (however frequently less marked) but the enlargement of IAC is anecdotical [3].

Many patients describe ataxia, aural fullness, headache, and facial hypesthesia happening more than 40% of the time after a hemorrhage. Non-HVS instances, on the other hand, report these symptoms in 10% of cases. Furthermore, there is a reported 10% risk of mortality in hemorrhagic instances, which is a significant consequence when compared to individuals with a 0.2% chance of death in non-HVS [5,6,10-13]. Although many symptoms are comparable in hemorrhagic and non-HVS, severe headache, acute hearing loss, facial hemiparesis, ataxia, diplopia, dizziness, nausea, and vomiting have been found to be more common in hemorrhagic VS (the so-called acoustic apoplexy syndrome) [5,11]. In the current case, it is likely that the abrupt intratumoral bleeding, which caused an increase in local pressure in the posterior fossa, aggravated a preexisting compression by the VS, exacerbating the symptomatic presentation.

The tumoral expansion rate seems to be a major risk factor for VS hemorrhagic transformation. Some point to cystic VS as more prone to bleed [14,15], due to higher expansion rates than the solid VS, but this remains uncertain, as others suggest that the appearance of a cystic component in VS may originate from prior subclinical intratumoral hemorrhage itself [15,16]. Other reported potential predisposing factors for hemorrhagic events in VS are anticoagulant therapy, pregnancy, heavy weightlifting, strenuous exercise, and hypertension [5,6]. Confronting our case with the suggested research, our patient’s tumor was solid and did not have any other potential risk factor for bleeding that we could account for. In APC tumors resulting in SAH, such as in HVS, early detection is important for the clinical outcome, since it may allow prompt treatment and avoid intracranial complications [5]. 

There are three standard surgical approaches to VS: retromastoid suboccipital (retrosigmoid), translabyrinthine, and middle fossa approach [6]. Irrespectively, intraoperative neuromonitoring is considered the standard of care in order to minimize additional injury to the facial nerve. In the case here described, the retrosigmoid approach was used allowing an adequate access and exposure to decompress the brainstem and cerebellum. The treatment of HVS is based on surgery, but radiation therapy, stereotactic surgery, and observation can also be a choice in selected cases [6]. Given that current guidelines recommend watchful waiting rather than surgical treatment, particularly for the geriatric population and “small” VSs, it seems reasonable to consider that unusual clinical presentations due to acute hemorrhage can become more common [12,17]. It is important to bear in mind that cystic, larger and rapidly growing tumors seem more prone to bleed. Also, the contribution of the neurosurgical team is often determinant to provide the best standard of care. Despite the improvement in facial paralysis, the current case presented ipsilateral cophosis six months after surgery. Pre-operative installed hearing loss seems to associate with poorer audiometric outcomes after surgical resection [18]. Hearing preservation remains a difficult endeavor, with rates of 20%-40% when utilizing the retrosigmoid technique and 30%-60% when using the middle fossa method approach [18]. This probably translates the sensory cochlear nerve's fragility in comparison to the considerably more strong motor facial nerve. Even when hearing is effectively maintained during surgery, only about half of the patients will keep useable hearing after 10 years [18]. Nevertheless, it is important to note that the natural evolution of VS inevitably includes an important deterioration in hearing. Not rarely, watchful waiting protocols also associate with poor audiometric outcomes [18]. Some authors suggest that active treatments such as surgery or radiotherapy can obtain better results than spontaneous outcomes when the aim is to preserve hearing [18].

The mechanism of bleeding in HVS is yet unknown. It might be attributed to the relatively rapid and uncontrolled growth of thin-walled, dilated, and sinusoidal vasculature within the tumor, especially when intracapsular such as in this case [19]. A better comprehension of the pathophysiology of HVS is halted by the rarity of this condition [19].

It is important to note that this case was first diagnosed at the Otorhinolaryngology emergency. In some hospitals, Otorhinolaryngology is the front-line specialty evaluating dizzy patients. This case highlights the pertinence of disseminating knowledge about rarer and potentially life-threatening complications of VS among Otorhinolaryngologists and other attending clinicians. Performing a gross audiologic evaluation with tuning forks, an additional audiometrical testing was determinant for raising the suspicion of a retrocochlear lesion, along with proper neurological examination. This article highlights the pertinence of timely VS diagnosis to allow for adequate follow-up and management. Also, it raises awareness of the importance of suspicion in the advent of intracranial complications.

## Conclusions

Despite being rare, hemorrhage can happen in patients with lesions of the APC. The clinician must be aware of its presenting signs in order to allow a timely diagnosis and prevent serious complications. Balancing the onco-functional effects of surgery is crucial to preserve facial nerves and nearby structures while alleviating symptoms and reducing recurrence.
